# Dental caries and fluorosis after 40 years of community water fluoridation in São Paulo, Brazil

**DOI:** 10.11606/s1518-8787.2025059007126

**Published:** 2026-01-09

**Authors:** Paulo Capel Narvai, Carlos Cesar da Silva Soares, Antonio Carlos Frias, José Leopoldo Ferreira Antunes

**Affiliations:** I Universidade de São Paulo. Faculdade de Saúde Pública. Departamento de Política, Gestão e Saúde. São Paulo, SP, Brasil; II Universidade de São Paulo. Faculdade de Odontologia. Departamento de Odontologia Social. São Paulo, SP, Brasil; III Universidade de São Paulo. Faculdade de Saúde Pública. Departamento de Epidemiologia. São Paulo, SP, Brasil

**Keywords:** Dental Caries, DMFT Index, Fluoridation, Dental Fluorosis, Evidence-Based Public Health

## Abstract

**OBJECTIVE:**

To analyze the non-exclusive impact of community water fluoridation on the prevalence of caries and dental fluorosis in schoolchildren in the municipality of São Paulo between 1986 and 2023.

**METHODS:**

Secondary data were used for the index age of 12 years, provided by the Ministry of Health, covering caries and dental fluorosis in different years, from 1986 to 2023. The degree of caries polarization was calculated using the Gini index. Two recognized risk factors for caries—low maternal schooling and per capita income—were obtained from the municipal human development index for 1991, 2000, and 2010, the years for which they are available. Exposure to community fluoridated water was verified by analyzing 49,515 water samples obtained between 1990 and 2021.

**RESULTS:**

Caries prevalence was 94.9% (95%CI: 92.7–97.1) in 1986; 68.1% (95%CI: 60.9–75.3) in 1996; 60.2% (95%CI: 54.2–66.3) in 2003; 47.6% (95%CI: 41.2–54.1) in 2010; and 46.3% (95%CI: 38.3–54.3) in 2023. The mean DMFT decreased from 6.47 (95%CI: 6.05–6.89) in 1986 to 2.37 (95%CI: 1.96–2.78) in 1996; 1.75 (95%CI: 1.48–2.02) in 2003; 1.40 (95%CI: 1.13–1.67) in 2010 and 1.51 (95%CI: 1.03–1.99) in 2023. The proportion of decayed teeth (“D” component of DMFT) was 58.5% in 1986; 33.3% in 1996; 32.3% in 2003; 51.2% in 2010, and 56.0% in 2023. Fluoride concentrations in the public water supply were adequate during the period in question, with values predominantly (98.0%) between 0.545 mgF/L and 0.944 mgF/L of water. The mean concentration was 0.663 mgF/L and the standard deviation was 0.167.

**CONCLUSION:**

The significant decline in the mean value of the DMFT index, in the order of 76.7% from 1986 to 2023, indicates the effectiveness of fluoridation, corroborating the scientific evidence related to its effectiveness and the continuity of this measure, especially considering the persistent difficulties in accessing dental care in the city of São Paulo.

## INTRODUCTION

The occurrence of dental caries in populations continues to be a major public health problem in several countries^1^. Although scientific evidence shows the multifactorial etiology of the disease, studies on its etiology have shown that the consumption of sugary products is a risk factor that plays a strategic role in the process leading to the pathological outcome. Nevertheless, there is widespread recognition that fermentable carbohydrates are low-cost sources of calories, and it is unlikely that their use will be controlled in contemporary societies. The main determinant of risk is believed to be not in the amount consumed, but the frequency of intake, which correlates positively with disease levels in populations^2^.

The virtual impossibility of restricting the consumption of cariogenic products on a population scale, due to socio-economic factors, notably cultural aspects, has forced decision-makers—involved in public policies to tackle dental caries—to look for protective factors that can counteract risk factors.

Scientific evidence on the properties of fluorides in caries prevention indicates their effectiveness in various forms and ways of use^3^. One of the means used to make fluoride available to the oral environment, at a population level, is the public health technology known as ‘fluoridation of the public water supply,” which, due to its characteristics, is considered a typical public health intervention, as it benefits everyone indistinctly, has proven efficacy by controlled studies, has a low relative cost, does not depend on actions carried out individually by the beneficiaries, does not require changes in the habits or attitudes of the population targeted by the intervention, and is safe for human health—the only known adverse effect is very mild degrees of dental fluorosis, with no functional or aesthetic implications^4^. Cases classified as moderate or severe are rare, or occur in a tiny percentage of the target population^5^. The measure is therefore recommended by scientists^6^, national^7^and international^8^health agencies, as well as the World Health Organization^9^.

São Paulo, a city in the southeastern region of Brazil, with a population estimated by the *Instituto Brasileiro de Geografia e Estatística* (IBGE — Brazilian Institute of Geography and Statistics) for 2025 of around 12 million inhabitants, is the largest city in the southern hemisphere and has many problems deriving from its urban characteristics, including the priority given to the automobile, excessive soil sealing, demographic emptying in consolidated areas, and the formation of peripheries lacking housing, infrastructure, services, and jobs. Its metropolitan area, comprising 38 other municipalities, will have a population of approximately 22 million inhabitants by 2025, ranking among the world’s five largest megacities.

In 1986, dental caries occurred in 95% of children in São Paulo, estimated for the index age of 12. The DMFT index, which expresses the number of permanent teeth (T) that are decayed (D), missing (M) or filled (F) at the time of examination, was 6.47 (95%CI: 6.12–6.82) in 1986^10^.

In 1982, during the elections for governor of the state of São Paulo, one of the candidates included in his healthcare campaign the introduction of community water fluoridation in municipalities that did not yet have it, including the capital, São Paulo, and other municipalities in the metropolitan region. Once elected, the governor ordered the fulfillment of his campaign promise at the beginning of 1983. Two years later, on October 31, 1985, the city of São Paulo began fluoridating its public water supply^10^. Since then, the measure has never been interrupted and, in October 2025, it will be 40 years since inhabitants have been continuously exposed to the fluorides present in public water supplies.

In Brazil, this public health measure began to be used in October 1953 in the municipality of Baixo Guandu, in the Rio Doce Valley, in Espírito Santo^11^. In 1974, the Brazilian National Congress passed Law 6.050/1974, making it compulsory to carry out the measure wherever there is a water treatment plant. In 2023, the National Oral Health Policy, instituted by Law No. 14.572/2023, included fluoridation of public water supplies among its ten guidelines, in the following terms: “to implement and maintain health surveillance actions for the fluoridation of public water supplies, mandatory under the terms of Law No. 6,050, of May 24, 1974, as well as complementary actions in places where they are necessary, and ensure that the public authorities have control over these actions” (our translation)^12^.

This study presents and discusses data on the epidemiology of caries and dental fluorosis at the index age of 12 in the city of São Paulo, assuming that community water fluoridation has an impact on population caries levels, helping to prevent the disease.

## METHODS

An epidemiological and health surveillance panel was built for the study period, using secondary data. For the caries and dental fluorosis outcomes, we used data obtained directly from the databases made available by the Ministry of Health, produced by epidemiological population surveys carried out between 1986 and 2023, which used the DMFT index and the Dean’s index for dental fluorosis, both recommended by the World Health Organization. Slight differences between the values presented in this article and the technical reports of these surveys are due to this option of calculating data from the bases.

The DMFT index makes it possible to measure the magnitude with which the disease is expressed in each person, since counting the number of D, M, and F teeth makes it possible to generate statistics considering different reference populations^13^. The index age of 12 years was used in the analysis. Caries experience (DMFT ≥ 1) was accepted as an estimator of prevalence, although its values include past experience with the disease, represented by the M and F components. The magnitudes in the different years analyzed were obtained by calculating means, confidence intervals (95%CI), and the percentage composition of the indicator.

The distribution of cases of the disease in the population was calculated using the Gini coefficient. The degree of polarization of the disease in the study population is described using Lorenz curves^14^, for the years 1986, 1996, 2003, 2010, and 2023. The epidemiological surveys that gave rise to the data varied in terms of type of investigation, design, sampling plan, and diagnostic criteria for the disease, but all produced estimates considered valid for the population of the city of São Paulo, for the index age used in this study^15^.

Since the mother’s low level of schooling and low per capita income are two recognized risk factors for caries^16^and since both are part of the municipal human development index (MHDI), the specific components for the evolution of education (MHDI-E), and income (MHDI-I) were extracted from the index for the years in which this indicator is available (1991, 2000, and 2010)^17^.

Given the importance of dental fluorosis in a context where there is exposure to multiple sources of fluoride, secondary data were used to analyze this dental formation anomaly. For the surveillance of water quality for human consumption, we used data from samples (n = 49,515) that included the fluoride parameter in the city of São Paulo, from 1990 to 2021. The results of the health surveillance panel were analyzed using the theoretical framework of Evidence-Based Public Health (EBPH)^18^.

EBPH is a set of rules that decision-makers on the implementation of public health policies should take into account when evaluating them, with reference to the need to simultaneously consider scientific evidence derived from evidence-based medicine, which is based on clinical criteria adopted in various types of research designs, notably randomized clinical trials, and the results obtained in the contextual conditions in which the policies are implemented, with the human, financial and technological resources available in each situation.

The aim of EBPH is to provide input for decision-making on the direction a given public health program or policy should take, whether to end it or continue it, in view of how resources should be allocated^19,20^. Brownson et al.^20^categorize two types of evidence, from the perspective of EBPH: a) type 1 evidence refers to risk, seeking to identify the magnitude, severity and possibilities of preventing the health problem, with the aim of informing “whether something should be done”; b) type 2 evidence seeks to identify the relative effectiveness of specific interventions with the potential to address and, if possible, solve the health problem, contributing to determining “what should be done.”

For Rychetnik et al.^21^, EBPH aims for an informed, explicit, and judicious use of evidence, derived from a variety of evaluative research, including the social sciences, originating from descriptive, taxonomic, analytical, interpretative, explanatory, and evaluative studies. Kemm^22^points out that EBPH is therefore grounded in both evidence-based medicine and other approaches to “scientific evidence,” since in “policy making evidence has always been interpreted broadly to cover all types of reasoned enquiry,” taking “communities rather than individuals as the unit of intervention” and recognizing “the importance of context,” since “frequently randomized controlled trials are not appropriate for study of public health interventions” that are intended to evaluate them.

## RESULTS

**Figure 1:**
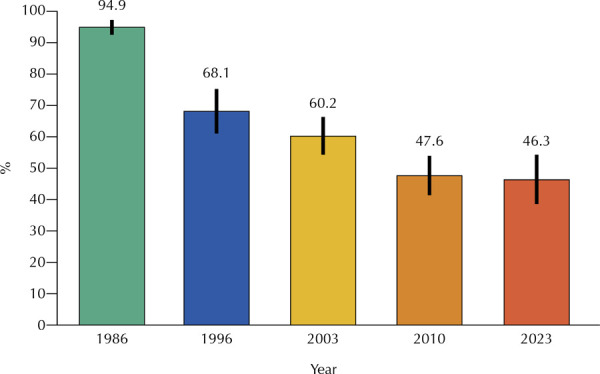
Caries prevalence at 12 years of age in different years in the city of São Paulo, SP.

Note: the size of the circle around the point estimate of the mean indicates the 95% confidence interval estimate of the population parameter.

Caries prevalence decreased from 94.9% (95%CI: 92.7–97.1) in 1986 to 46.3% (95%CI: 38.3–54.3) in 2023 (Figure 1).

**Figure 2:**
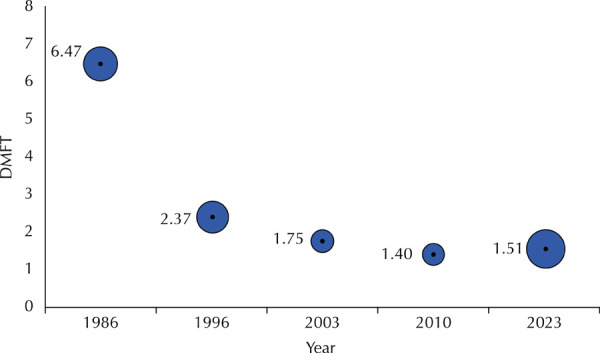
DMFT index at 12 years of age in different years in the city of São Paulo, SP.

The magnitude of caries experience, as measured by the DMFT index, followed the decline observed in the prevalence of the disease, decreasing by 76.7% (Figure 2).

**Figure 3:**
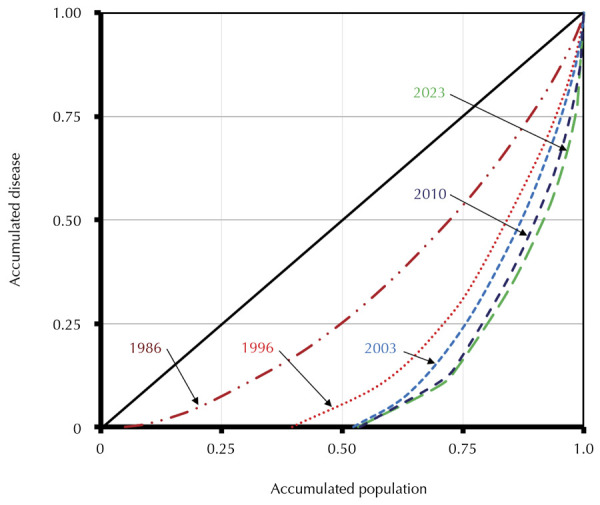
Inequality in the distribution of caries at 12 years of age in different years in the city of São Paulo, SP.

Although both the prevalence and magnitude of caries showed a significant decrease in the study population, the concentration of cases in the population groups where the disease occurs most severely increased, thus increasing inequality in its population distribution, as shown by the Lorenz curves for the Gini coefficient in Figure 3.

**Figure 4:**
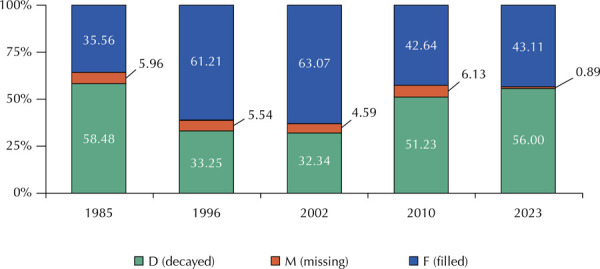
Percentage composition of the DMFT index at 12 years of age in different years in the city of São Paulo, SP.

Figure 4 shows that the proportion of decayed teeth (component “D” of the DMFT) was 58.48% in 1985; 33.25% in 1996; 32.34% in 2002; 51.23% in 2010, and 56.00% in 2023.

**Figure 5:**
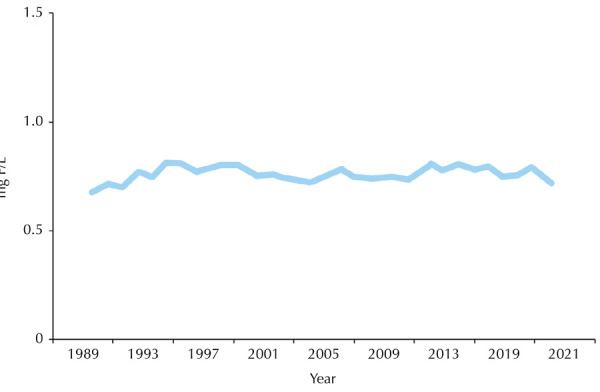
Mean values of fluoride concentrations (mg/L) obtained from samples (n = 49,515) of public water supply in the city of São Paulo, from 1989 to 2021.

Note: the size of the circle around the point estimate of the percentage indicates the 95% confidence interval estimate of the population parameter.

Figure 5 shows that the study population was exposed to fluoride concentrations in the public water supply with values predominantly (97.97%) between 0.545 mgF/L and 0.944 mgF/L of water. Between 1990 and 2021, the mean concentration was 0.663 mgF/L, with a standard deviation of 0.167. The fluctuations in concentration were insignificant, corresponding to 2.03% of the 49,515 samples collected by the Health Surveillance Coordination (COVISA) of the São Paulo Municipal Health Department, at different points in the territory of the municipality of São Paulo.

**Figure 6:**
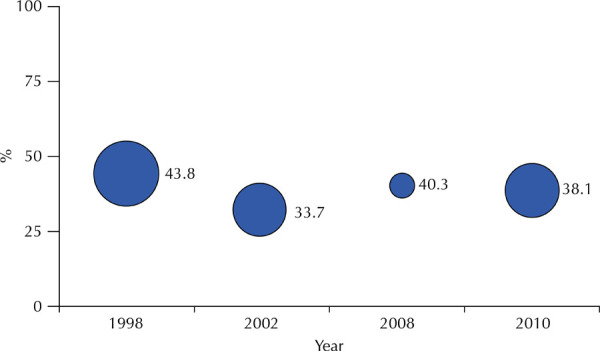
Prevalence of dental fluorosis and 95% confidence intervals in 12-year-old children. Municipality of São Paulo, 1998, 2002, 2008, and 2010.

Figure 6 shows the prevalence of dental fluorosis, assessed by the Dean index^13^, in 1998, 2002, 2008, and 2010 in 12-year-old children in São Paulo. The situation remained stable over the period analyzed. The 2023 national epidemiological survey did not assess dental fluorosis at a population level. The article^23^ from which Figure 5 was taken shows that, together, the “very mild + mild” categories, considered functionally and aesthetically irrelevant, registered 38.4% (95%CI: 30.3–47.6) in 1998; 32.1% (95%CI: 26.6–38.2) in 2002; 38.0% (95%CI: 36.5–39.5) in 2008, and 36.4% (95%CI: 30.4–42.7) in 2010. In 1998, there was one case of severe fluorosis in the sample used and, in the other years, no cases were observed.

## DISCUSSION

### Health Surveillance Panel

The mean values and confidence intervals of the DMFT estimates indicate that there was a statistically significant difference between 1986 and 1996 (p < 0.001). However, in the first decades of the 21st century, both the prevalence and magnitude of the disease (p = 0.112) remained stable in the population. The significant decline in the mean value of the DMFT index, of around 76.7% from 1986 to 2023, reveals the effectiveness of fluoridation, corroborating the scientific evidence related to its efficacy^24,25^. However, given the risk of interrupting its implementation in São Paulo, brought about by the contemporary debate in Brazil and around the world about this technology, it is appropriate to ask about the impact of this possible interruption on population caries levels.

Due to the characteristics and purpose of the epidemiological panel presented in this study, it is not possible to measure the extent to which exposure to community fluoridated water has contributed to reducing the prevalence and magnitude of caries in the study population. It is widely accepted among public health experts that the use of fluoridated toothpastes and changes in oral health programs during this period are factors associated with community water fluoridation for the change recorded in the epidemiology of caries. Based on studies carried out in Brazil, the preventive fraction that can be attributed to fluoridation ranges between 34% and 47%^26,27^.

However, from 1986 to 2023, the share of the “D” component in the composition of the DMFT value fluctuated in the different years, increasing in 2023 (56.0%) to the proportion recorded in 1986 (58.5%), indicating that difficulties of access to dental care persist in the city of São Paulo.

The study population was demonstrably continuously exposed to adequate fluoride concentrations in the public water supply during the period under analysis, which is in line with what is known about the importance of not interrupting this measure over time, so that its effectiveness is not compromised^25,28,29^. The adverse effect of dental fluorosis did not constitute a relevant health problem in the population, as mild or very mild levels of the anomaly predominated, with no aesthetic or functional impairment, similar to what is observed in other locations in the Brazilian context^30,31^.

The effectiveness of community water fluoridation technology has contributed to the significant decline in the mean value of the DMFT index. Associated with this decline, there has also been a decrease in the share of the “missing teeth” component in the total value of the DMFT index, which oscillates between 4.59% and 6.13% and in 2023 was only 0.89%. This result is in line with that reported by Crocombe et al.^32^and Dickson-Swift et al.^33^, among others.

### Evidence-based Public Health

Noting that historically public health has always been more evidence-based than other health sciences, particularly with regard to disease prevention and health protection, and warning that evidence on the etiological role of agents and the potential effectiveness of actions must be available before deciding to implement a public health intervention, Jenicek^34^proposed in 1997 a set of requirements for analyses based on EBPH.

Seven items can be highlighted from this set of requirements, listed as: (a) formulating a clear question based on a public health problem; (b) gathering scientific evidence; (c) gathering and analyzing evidence; (d) basing decision-making on the best evidence, with reference to public health conditions; (e) linking evidence with experience, knowledge and practice in public health; (f) implementing useful evidence in public health practice, i.e. in public policy or a specific health program; and (g) evaluating, based on evidence, the implementation and overall performance of the policy, including public health professionals.

In this study, it was observed that the implementation of community water fluoridation over 40 years meets Jenicek’s seven requirements^34^, because: a) the public health problem represented by an epidemiological profile characterized by high prevalence and magnitude of dental caries showed a relevant change in the implementation period analyzed, positively impacting both prevalence and magnitude; b) scientific evidence is available on the efficacy, effectiveness, and efficiency of the action implemented; c) the data presented in the health surveillance panel is consistent, proving exposure and the impact of exposure, although not exclusive; d) the decision to use public health technology to prevent dental caries was based on the best evidence available at the time, with reference to studies on public health conditions in the population in the context of the mid-1980s; e) the experience, knowledge and practice in public health with community water fluoridation in Brazil and in other countries constituted a solid basis for decision-making; f) the implementation of community water fluoridation was accompanied by water quality surveillance practices for human consumption, with emphasis on fluoride concentrations; and g) the implementation and overall performance of the policy has proven to be effective in tackling and positively impacting the public health problem represented by dental caries. For these reasons, it can be said that, given the recognized effectiveness of community water fluoridation as a public health measure^24,25^, and the fact that the application of this technology in the city of São Paulo has been effective, even though dental caries is a disease associated with more than 70 risk factors, there should be no consideration of discontinuing its implementation in the city.

Among those in Brazil and around the world who advocate discontinuing the use of this public health measure, it is argued that it is not possible to attribute to community water fluoridation alone the preventive effects indicated by the data presented in this study. In fact, the epidemiological profile of caries presented in this study may have been affected by other protective factors, as suggested. However, no studies were identified that focused on the set of risk factors for dental caries in the population under analysis.

As the association between community water fluoridation and lower levels of prevalence and magnitude of the disease is well established^35^, we hypothesize that the most plausible explanation for the significant declines in the prevalence and magnitude of the disease in the population of São Paulo stems above all from two protective factors whose scientific evidence is consistent: public water supply (fluoridated in São Paulo since 1985) and toothpastes (with most brands fluoridated since 1988). Increased access to these protective factors was combined in Brazil, and in the city of São Paulo, with changes in the oral health programs in the public health system, which began to emphasize educational actions, related, among other things, to increasing and adapting tooth brushing and decreasing the frequency of ingestion of sugary products^36^. It should be noted, however, that according to data provided by the Atlas of Human Development in Brazil^17^, the MHDI in the city of São Paulo has evolved positively: from 0.626 (medium) in 1991, to 0.733 (high) in 2000 and 0.807 (very high) in 2010. There were improvements in education and income levels, as the MHDI-E rose from 0.421 in 1991 to 0.614 in 2000 and 0.725 in 2010, while the MHDI-I was 0.784 in 1991, 0.807 in 2000, and 0.843 in 2010. Therefore, these factors can be considered as relevant contributors to the decline in the prevalence and magnitude of caries in the population analyzed.

In this regard, Brownson et al.^20^state that the art of decision making involving a public policy or a public health program, based on EBPH, usually involves knowing what information is important, because “unlike solving a math problem, significant decisions in public health must balance science and art, since evidence-based decision making often involves choosing one alternative from among a set of rational choices.” Notably, It should be recognized that rational choices are not politically and ideologically neutral, but informed by values and beliefs that give them different rationalities, all of which are legitimate.

The potential and limitations of this article are those inherent in the EBPH approach. It tacitly presents potential for understanding the role fluoridation has played in the 40 years since its implementation in the city. Its limitations derive from the fact that it deals with a public health intervention that has been underway for four decades. The article explores the period of the first of these four decades, discussing the findings with the relevant literature, and presents possibilities for understanding the subsequent three decades, placing this intervention in the historical context in which it was developed and presenting the problem that its eventual interruption could cause.

## CONCLUSION

Considering the persistent difficulties in accessing dental care in the city of São Paulo and the risk of fluoridation being discontinued in São Paulo, brought about by the contemporary debate on the subject in Brazil and around the world, it is appropriate to ask, as we have explained in this article, about the impact of this possible discontinuation on population levels of dental caries.

Pressures to discontinue community water fluoridation in the city of São Paulo and other Brazilian locations^37^, based on opinions devoid of scientific evidence, should be rejected by decision-makers in the field of public policy. To this end, the EBPH perspective presented in this article can contribute to supporting the continuation of this preventive measure in the city.

As public health technology, community water fluoridation is not a dogma^38^, nor is it closed to scientific research. It should be evaluated with due scientific rigor and, if necessary, discontinued—as some countries do. However, a decision of this nature must be made taking into account its complexity. Religious, economic, epidemiological and health motivations must be carefully analyzed as the basis for such a decision.

## Data Availability

The study was conducted using data collected from databases made publicly available by the Ministry of Health. Any interested party can access these databases by contacting the Ministry of Health’s General Coordination of Oral Health at cosab@saude.gov.br.
