# Supplementary material for: Dental caries and fluorosis after 40 years of community water fluoridation in São Paulo, Brazil
Source: Rev Saude Publica. 2026 Jan 9;59:e47. doi: 10.11606/s1518-8787.2025059007126 (PMC12713525; doi:10.11606/s1518-8787.2025059007126)
Supplement: Translation

# Translation: Cárie e fluorose dentária após 40 anos de fluoretação da água em São Paulo, Brasil

**Authors:** Paulo Capel Narvai, Carlos Cesar da Silva Soares, Antonio Carlos Frias, José Leopoldo Ferreira Antunes

**Affiliations:** I Universidade de São Paulo. Faculdade de Saúde Pública. Departamento de Política, Gestão e Saúde. São Paulo, SP, Brasil; II Universidade de São Paulo. Faculdade de Odontologia. Departamento de Odontologia Social. São Paulo, SP, Brasil; III Universidade de São Paulo. Faculdade de Saúde Pública. Departamento de Epidemiologia. São Paulo, SP, Brasil

**Keywords:** Cárie Dentária, Índice CPOD, Fluoretação, Fluorose Dentária, Saúde Pública Baseada em Evidência

## Abstract

**OBJETIVO:**

Analisar o impacto, não exclusivo, da fluoretação da água na prevalência de cárie e fluorose dentária em escolares do município de São Paulo entre 1986 e 2023.

**MÉTODOS:**

Foram utilizados dados secundários para a idade-índice de 12 anos, disponibilizados pelo Ministério da Saúde, contemplando cárie e fluorose dentária em diferentes anos, abrangendo o período de 1986 a 2023. O grau de polarização da cárie foi calculado pelo índice de Gini. Dois reconhecidos fatores de risco para cárie, as baixas escolaridade da mãe e renda *per capita*, foram obtidos do índice de desenvolvimento humano municipal para 1991, 2000 e 2010, anos para os quais estão disponíveis. A exposição à água fluoretada foi comprovada pela análise de 49.515 amostras da água obtidas no período de 1990 a 2021.

**RESULTADOS:**

A prevalência de cárie foi de 94,9% (IC95% 92,7–97,1) em 1986; 68,1% (IC95% 60,9–75,3) em 1996; 60,2% (IC95% 54,2–66,3) em 2003; 47,6% (IC95% 41,2–54,1) em 2010; e 46,3% (IC95% 38,3–54,3) em 2023. A média CPOD regrediu de 6,47 (IC95% 6,05–6,89) em 1986 para 2,37 (IC95% 1,96–2,78) em 1996; 1,75 (IC95% 1,48–2,02) em 2003; 1,40 (IC95% 1,13–1,67) em 2010 e 1,51 (IC95% 1,03–1,99) em 2023. A proporção de dentes cariados (componente “C” do CPOD) registrou 58,5% em 1986; 33,3% em 1996; 32,3% em 2003; 51,2% em 2010 e 56,0% em 2023. As concentrações de fluoretos na água de abastecimento público foram adequadas no período considerado, com valores predominantemente (98,0%) situados entre 0,545 mgF/L e 0,944 mgF/L de água. A concentração média foi de 0,663 mgF/L e o desvio-padrão de 0,167.

**CONCLUSÃO:**

O expressivo declínio no valor médio do índice CPOD, da ordem 76,7% entre 1986 e 2023, indica a efetividade da fluoretação, corroborando a evidência científica relacionada com sua eficácia e a continuidade dessa medida, sobretudo, considerando-se as dificuldades persistentes de acesso a cuidados odontológicos assistenciais na cidade de São Paulo.

## INTRODUÇÃO

A ocorrência da cárie dentária em populações segue sendo um importante problema de saúde pública em vários países^1^. Embora evidências científicas demonstrem o caráter multifatorial da doença, estudos sobre sua etiologia têm comprovado que o consumo de produtos açucarados é um fator de risco que ocupa função estratégica no processo que conduz ao desfecho patológico. Não obstante, há amplo reconhecimento de que carboidratos fermentáveis são fontes calóricas de baixo custo, sendo improvável, nas sociedades contemporâneas, controlar seu uso. Pondera-se que o principal determinante do risco não é a quantidade consumida, mas a frequência de ingestão, que se correlaciona positivamente com os níveis da doença nas populações^2^.

A virtual impossibilidade de restringir o consumo de produtos cariogênicos em escala populacional, decorrente de fatores socioeconômicos, notadamente aspectos culturais, vem impondo a tomadores de decisão – envolvidos com políticas públicas para enfrentamento da cárie dentária – buscar por fatores de proteção que possam se contrapor aos fatores de risco.

As evidências científicas sobre as propriedades dos fluoretos na prevenção da cárie indicam sua eficácia, sob várias formas e modos de utilização^3^. Um dos meios utilizados para disponibilizar fluoretos para o ambiente bucal, em nível populacional, é a tecnologia de saúde pública conhecida como “fluoretação da água de abastecimento público”, a qual, por suas características, é considerada uma típica intervenção de saúde pública, pois beneficia a todos indistintamente, tem eficácia comprovada por estudos controlados, apresenta baixo custo relativo, não depende de ações realizadas individualmente pelos beneficiados, não requer mudanças de hábitos ou atitudes da população-alvo da intervenção, sendo segura para a saúde humana – o único efeito adverso conhecido são graus muito leves de fluorose dentária, sem implicações funcionais ou estéticas^4^. Casos classificados como moderado ou grave são raros, ou ocorrem em porcentagem ínfima da população-alvo^5^. Assim, a medida é recomendada por cientistas^6^, organismos nacionais^7^ e internacionais^8^ de pesquisa em saúde, bem como a Organização Mundial da Saúde^9^.

São Paulo, município da região sudeste do Brasil, com uma população estimada pelo Instituto Brasileiro de Geografia e Estatística (IBGE) para 2025 de cerca de 12 milhões de habitantes, é a maior cidade do hemisfério sul, apresentando muitos problemas derivados de suas características urbanas, entre os quais a prioridade para o automóvel, a excessiva impermeabilização do solo, o esvaziamento demográfico em áreas consolidadas, a formação de periferias carentes de habitação, infraestrutura, serviços e empregos. Sua região metropolitana, integrada por outros 38 municípios, comporta uma população de aproximadamente 22 milhões de habitantes em 2025, situando-se entre as cinco megametrópoles mundiais.

Em 1986, a cárie dentária ocorria em 95% das crianças de São Paulo, estimada para a idade-índice de 12 anos. O índice CPOD, cujo valor expressa o número de dentes permanentes (D) que, no momento do exame, estão cariados (C), extraídos (P) ou restaurados (O), registrava 6,47 (IC95% 6,12–6,82) em 1986^10^.

Em 1982, nas eleições para o governo do estado de São Paulo, um dos candidatos fez constar em sua proposta de campanha eleitoral, para a saúde, a introdução da fluoretação da água nos municípios que não a utilizavam, incluindo a capital São Paulo e os demais municípios da região metropolitana. Eleito, o governador determinou, no início de 1983, que se cumprisse a promessa de campanha. Dois anos depois, em 31 de outubro de 1985, a cidade de São Paulo deu início à fluoretação das águas^10^. Desde então, a medida jamais foi interrompida e, em outubro de 2025, completam-se 40 anos de exposição continuada dos habitantes aos fluoretos presentes na água de abastecimento público.

No Brasil, essa medida de saúde pública começou a ser utilizada em outubro de 1953 no município de Baixo Guandu, no Vale do Rio Doce, no Espírito Santo^11^. Em 1974, o Congresso Nacional brasileiro aprovou a Lei nº 6.050/1974, tornando obrigatória a execução da medida onde houver estação de tratamento de água. Em 2023, a Política Nacional de Saúde Bucal, instituída pela Lei nº 14.572/2023, incluiu a fluoretação das águas de abastecimento público entre suas dez diretrizes, nos seguintes termos: “implantar e manter ações de vigilância sanitária de fluoretação das águas de abastecimento público, obrigatória nos termos da Lei nº 6.050, de 24 de maio de 1974, bem como ações complementares nos locais em que se fizerem necessárias, e assegurar ao poder público controle sobre essas ações”^12^.

Neste artigo são apresentados e discutidos dados sobre a epidemiologia da cárie e da fluorose dentária na idade-índice de 12 anos na cidade de São Paulo, admitindo-se que a fluoretação da água impacta os níveis populacionais de cárie, contribuindo para prevenir a doença.

## MÉTODOS

Foi construído um painel contemplando aspectos de vigilância epidemiológica e sanitária para o período do estudo, utilizando-se dados secundários. Para o desfecho cárie e fluorose dentária, foram usados dados apurados diretamente das bases de dados disponibilizadas pelo Ministério da Saúde, produzidos por inquéritos populacionais epidemiológicos realizados no período de 1986 a 2023, que empregaram o índice CPOD e o índice de Dean para fluorose dentária, ambos preconizados pela Organização Mundial da Saúde. Pequenas diferenças entre os valores apresentados neste artigo e os relatórios técnicos desses inquéritos decorrem dessa opção de apurar dados a partir das bases.

O índice CPOD possibilita dimensionar a magnitude com que a doença se expressa em cada pessoa, pois a contagem do número de dentes permanentes C, P e O permite gerar estatísticas considerando diferentes populações de referência^13^. A idade-índice de 12 anos foi empregada na análise. A experiência de cárie (CPOD ≥ 1) foi admitida como um estimador das prevalências, ainda que carregando em seus valores a experiência passada com a doença, representada pelos componentes P e O. As magnitudes nos diferentes anos analisados foram obtidas calculando-se médias, intervalos de confiança (IC95%) e composição percentual do indicador.

A distribuição dos casos da doença na população foi dimensionada utilizando o coeficiente de Gini. O grau de polarização da doença na população de estudo é descrito por meio de curvas de Lorenz^14^, para os anos de 1986, 1996, 2003, 2010 e 2023. Os inquéritos epidemiológicos que deram origem aos dados variaram quanto ao tipo de investigação, delineamento, plano amostral e critério diagnóstico para a doença, mas todos produziram estimativas consideradas válidas para a população da cidade de São Paulo, para a idade-índice utilizada neste estudo^15^.

Como o baixo nível de escolaridade da mãe e a baixa renda *per capita* são dois reconhecidos fatores de risco para a cárie^16^ e sendo que ambos participam do índice de desenvolvimento humano municipal (IDHM), foram extraídos do índice os componentes específicos para a evolução da escolaridade (IDHM-E) e da renda (IDHM-R) para os anos em que esse indicador está disponível (1991, 2000 e 2010)^17^.

Tendo em vista a importância da fluorose dentária em contexto em que há exposição a múltiplas fontes de fluoretos, foram utilizados dados secundários para essa anomalia de formação dentária. Para a vigilância da água para consumo humano, utilizaram-se dados de amostras (n = 49.515) que contemplavam o parâmetro fluoreto na cidade de São Paulo, no período de 1990 a 2021. Os resultados do painel de vigilância em saúde foram analisados tendo como referencial teórico a Saúde Pública Baseada em Evidência (SPBE)^18^.

A SPBE constitui um conjunto de regras que tomadores de decisão sobre a implementação de políticas públicas de saúde devem levar em consideração para avaliá-las, tendo como referência a necessidade de, simultaneamente, considerar as evidências científicas derivadas da medicina baseada em evidência, que se fundamenta em critérios clínicos adotados em vários tipos de delineamentos de pesquisa, notadamente os ensaios clínicos randomizados e os resultados obtidos nas condições contextuais em que as políticas são executadas, com os recursos humanos, financeiros e tecnológicos disponíveis em cada situação.

O objetivo da SPBE é produzir subsídios para a tomada de decisão sobre o rumo que deve ser dado a um determinado programa ou política pública de saúde, encerrando-o ou dando-lhe prosseguimento, tendo em vista o modo como os recursos devem ser alocados^19,20^. Brownson et al.^20^ categorizam dois tipos de evidência, na perspectiva da SPBE: a) a evidência do tipo 1 se refere ao risco, buscando identificar a magnitude, a gravidade e as possibilidades de prevenir o problema de saúde, com o objetivo de informar “se algo deve ser feito”; b) a evidência do tipo 2 busca identificar a efetividade relativa de intervenções específicas com potencial para enfrentar e, se possível, resolver o problema de saúde, contribuindo para determinar “o que deve ser feito”.

Para Rychetnik et al.^21^, a SPBE almeja um uso informado, explícito e criterioso de evidências, derivado de uma variedade de pesquisas avaliativas, incluindo as ciências sociais, com origem em estudos descritivos, taxonômicos, analíticos, interpretativos, explicativos e avaliativos. Kemm^22^ assinala que a SPBE busca, portanto, seu fundamento tanto na medicina baseada em evidência quanto em outras abordagens sobre “evidência científica”, uma vez que na “formulação de políticas, as evidências sempre foram interpretadas de forma ampla, abrangendo todos os tipos de investigação fundamentada”, tomando “comunidades, em vez de indivíduos, como unidade de intervenção” e reconhecendo “a importância do contexto”, pois “frequentemente, ensaios clínicos randomizados não são apropriados para o estudo de intervenções em saúde pública”, que tenham o propósito de avaliá-las.

## RESULTADOS

A prevalência de cárie diminuiu de 94,9% (IC95% 92,7–97,1) em 1986 para 46,3% (IC95% 38,3–54,3) em 2023 (Figura 1).

**Figura 1:** Prevalência de cárie aos 12 anos de idade em diferentes anos na cidade de São Paulo, SP.

Nota: o tamanho do círculo em torno da estimativa pontual da média indica a estimativa por intervalo de confiança de 95% do parâmetro populacional.

A magnitude da experiência de cárie, aferida pelo índice CPOD, acompanhou o declínio observado na prevalência da doença, diminuindo 76,7% (Figura 2).

**Figura 2:** Índice CPOD aos 12 anos de idade em diferentes anos na cidade de São Paulo, SP.

Embora tanto a prevalência quanto a magnitude da cárie tenham apresentado expressiva diminuição na população de estudo, acentuou-se a concentração de casos nos grupos populacionais em que a doença ocorre com maior gravidade, aumentando, portanto, a desigualdade em sua distribuição populacional, conforme mostram as curvas de Lorenz para o coeficiente de Gini, na Figura 3.

**Figura 3:** Desigualdade na distribuição de cáries aos 12 anos de idade em diferentes anos na cidade de São Paulo, SP.

A Figura 4 mostra que a proporção de dentes cariados (componente “C” do CPOD) registrou 58,48% em 1985; 33,25% em 1996; 32,34% em 2002; 51,23% em 2010 e 56,00% em 2023.

**Figura 4:** Composição percentual do índice CPOD aos 12 anos de idade em diferentes anos na cidade de São Paulo, SP.

A Figura 5 mostra que a população de estudo esteve exposta a concentrações de fluoretos na água de abastecimento público com valores predominantemente (97,97%) situados entre 0,545 mgF/L e 0,944 mgF/L de água. Entre 1990 e 2021, a concentração média foi de 0,663 mgF/L, com desvio-padrão de 0,167. As flutuações de concentração foram insignificantes, correspondendo a 2,03% das 49.515 amostras coletadas pela Coordenação de Vigilância Sanitária, da Secretaria Municipal de Saúde de São Paulo, em diferentes pontos do território do município de São Paulo.

**Figura 5:** Valores médios das concentrações de fluoreto (mg/L) obtidos em amostras (n = 49.515) da água de abastecimento público na cidade de São Paulo, no período de 1989 a 2021.

Nota: o tamanho do círculo em torno da estimativa pontual da porcentagem indica a estimativa por intervalo de confiança de 95% do parâmetro populacional.

A Figura 6 mostra a prevalência de fluorose dentária, avaliada pelo índice de Dean^13^, nos anos de 1998, 2002, 2008 e 2010 em crianças de 12 anos de idade, em São Paulo. A situação é estacionária no período analisado. O inquérito epidemiológico nacional de 2023 não avaliou a fluorose dentária em nível populacional. No artigo^23^ de onde foi extraída a Figura 5 consta que, juntas, as categorias “muito leve + leve”, consideradas irrelevantes funcional e esteticamente, registraram 38,4% (IC95% 30,3–47,6) em 1998; 32,1% (IC95% 26,6–38,2) em 2002; 38,0% (IC95% 36,5–39,5) em 2008 e 36,4% (IC95% 30,4–42,7) em 2010. Em 1998, houve um caso de fluorose grave na amostra utilizada e, nos demais anos, nenhum caso foi observado.

**Figura 6:** Prevalência de fluorose dentária e intervalos de confiança de 95% em crianças de 12 anos de idade. Município de São Paulo, 1998, 2002, 2008 e 2010.

## DISCUSSÃO

### Painel de Vigilância em Saúde

Os valores médios e os intervalos de confiança das estimativas do CPOD indicam que houve diferença estatisticamente significativa entre 1986 e 1996 (p < 0,001). Contudo, nas primeiras décadas do século XXI, tanto a prevalência quanto a magnitude da doença (p = 0,112) se mostraram estáveis na população. O expressivo declínio no valor médio do índice CPOD, da ordem de 76,7% entre 1986 e 2023, revela a efetividade da fluoretação, corroborando a evidência científica relacionada com sua eficácia^24,25^. Contudo, frente ao risco de interrupção da sua implementação em São Paulo, trazido pelo debate contemporâneo, no Brasil e no mundo, acerca dessa tecnologia, é cabível indagar sobre o impacto dessa eventual interrupção sobre os níveis populacionais de cárie.

Não é possível, pelas características e finalidade do painel epidemiológico apresentado neste estudo, dimensionar o quanto a exposição à água fluoretada contribuiu para a redução da prevalência e da magnitude da ocorrência de cárie na população de estudo. Admite-se, entre especialistas em saúde pública, que o uso de cremes dentais fluoretados e as mudanças nos programas de saúde bucal nesse período são fatores associados à fluoretação das águas para a mudança registrada na epidemiologia da cárie. Com base em estudos realizados no Brasil, a fração preventiva que pode ser atribuída à fluoretação oscila entre 34% e 47%^26,27^.

Entretanto, entre 1986 e 2023, a participação do componente “C” na composição do valor do CPOD oscilou nos diferentes anos, aumentando em 2023 (56,0%) à proporção registrada em 1986 (58,5%), indicando que persistem, na cidade de São Paulo, dificuldades de acesso a cuidados odontológicos assistenciais.

A população de estudo esteve, comprovadamente, exposta continuamente a concentrações adequadas de fluoretos na água de abastecimento público, no período sob análise, o que vai ao encontro do que se sabe sobre a importância da não interrupção dessa medida ao longo do tempo, para que sua eficácia não seja comprometida^25,28,29^. O efeito adverso da fluorose dentária não se constituiu em problema de saúde relevante na população, pois predominaram níveis muito leves ou leves da anomalia, sem comprometimento estético ou funcional, de modo similar ao que se observa em outras localidades no contexto brasileiro^30,31^.

A efetividade da tecnologia de fluoretação da água contribuiu para o expressivo declínio no valor médio do índice CPOD. Associado a esse declínio, observa-se diminuição também na participação do componente “dentes perdidos” no valor total do índice CPOD, que oscila entre 4,59% e 6,13% e que, em 2023, registra apenas 0,89%. Esse resultado está em consonância com o relatado por Crocombe et al.^32^ e Dickson-Swift et al.^33^, dentre outros.

### Saúde Pública Baseada em Evidência

Constatando que historicamente a saúde pública sempre foi mais baseada em evidências do que outras ciências da saúde, particularmente no que se refere à prevenção de doenças e à proteção da saúde, e alertando que evidências sobre o papel etiológico dos agentes e as possibilidades de efetividade das ações devem estar disponíveis, antes que se decida pela execução de uma intervenção de saúde pública, Jenicek ^34^ propôs em 1997 um conjunto de requisitos para análises que tenham como referência a SPBE.

Desse conjunto podem ser destacados sete itens, enunciados como: a) formular uma pergunta clara a partir de um problema de saúde pública; b) reunir evidências científicas; c) reunir e analisar comprovações; d) embasar a tomada de decisão nas melhores evidências, tendo como referência as condições da saúde pública; e) vincular evidências com experiência, conhecimentos e prática em saúde pública; f) implementar as evidências úteis na prática de saúde pública, ou seja, na política pública ou programa de saúde específico; e g) avaliar, com base em evidências, a implementação e o desempenho geral da política, incluindo os profissionais de saúde pública.

Neste estudo, observa-se que a implementação da fluoretação da água de abastecimento, durante 40 anos, contempla os sete requisitos de Jenicek^34^, pois: a) o problema de saúde pública representado por um perfil epidemiológico caracterizado por altas prevalência e magnitude da cárie dentária apresentou mudança relevante no período de implementação analisado, impactando positivamente tanto a prevalência quanto a magnitude; b) estão disponíveis evidências científicas sobre a eficácia, efetividade e eficiência da ação implementada; c) os dados apresentados no painel de vigilância em saúde são consistentes, comprovando a exposição e o impacto da exposição, ainda que não exclusivos; d) a tomada de decisão sobre o uso da tecnologia de saúde pública para prevenir a cárie dentária foi embasado nas melhores evidências disponíveis à época, tendo como referência estudos sobre as condições da saúde pública na população, no contexto de meados dos anos 1980; e) a experiência, conhecimentos e prática em saúde pública com a fluoretação das águas no Brasil e em outros países constituía um sólido fundamento para a tomada de decisão; f) a implementação da fluoretação da água foi acompanhada por práticas de vigilância da qualidade da água para consumo humano, com ênfase para as concentrações do parâmetro fluoreto; e g) a implementação e o desempenho geral da política vêm mostrando-se efetivos para enfrentar e impactar positivamente o problema de saúde pública representado pela cárie dentária. Por essas razões, é possível afirmar que, com a reconhecida eficácia da fluoretação da água como uma medida de saúde pública^24,25^, e a constatação de que a aplicação dessa tecnologia na cidade de São Paulo vem sendo efetiva, embora a cárie dentária seja uma enfermidade que se associa a mais de 70 fatores de risco, não se deveria sequer cogitar a descontinuidade de sua implementação.

Entre os que advogam, no Brasil e no mundo, a interrupção do uso dessa medida de saúde pública, argumenta-se não ser possível atribuir à fluoretação da água, isoladamente, os efeitos preventivos que os dados apresentados neste estudo indicam. Com efeito, o perfil epidemiológico da cárie apresentado neste estudo pode ter sido afetado por outros fatores de proteção, conforme cogitado. Porém, não foram identificados estudos que tenham tomado como objeto o conjunto dos fatores de risco para cárie dentária na população sob a presente análise.

Como é bem estabelecida a associação entre fluoretação da água com menores níveis de prevalência e magnitude da doença^35^, aventa-se a hipótese de que a explicação mais plausível para os expressivos declínios na prevalência e magnitude da doença na população paulistana decorrem, sobretudo, de dois fatores de proteção cuja evidência científica é consistente: a água de abastecimento público (fluoretada em São Paulo desde 1985) e os cremes dentais (com a maioria das marcas fluoretadas a partir de 1988). A ampliação do acesso a esses fatores de proteção se combinou no Brasil, e na cidade de São Paulo, com mudanças nos programas de saúde bucal no sistema público de saúde, que passaram a enfatizar ações educativas, relacionadas, dentre outros aspectos, com o incremento e adequação da escovação dental e com a diminuição da frequência da ingestão de produtos açucarados^36^. Cabe registrar, porém, que, de acordo com dados disponibilizados pelo Atlas do Desenvolvimento Humano no Brasil^17^, o IDHM na cidade de São Paulo evoluiu positivamente: de 0,626 (médio) em 1991, para 0,733 (alto) em 2000 e 0,807 (muito alto) em 2010. Houve melhorias nos níveis de escolaridade e renda, pois o IDHM-E evoluiu de 0,421 em 1991, para 0,614 em 2000 e 0,725 em 2010, enquanto o IDHM-R registrou 0,784 em 1991, 0,807 em 2000, alcançando 0,843 em 2010. Portanto, esses fatores podem ser admitidos como coadjuvantes relevantes nos declínios da prevalência e magnitude da cárie na população analisada.

A esse respeito, Brownson et al.^20^ afirmam que a arte da tomada de decisão envolvendo uma política pública ou um programa de saúde pública, com base em SPBE, geralmente envolve saber quais informações são importantes, pois “ao contrário da resolução de um problema matemático, as decisões significativas em saúde pública devem equilibrar ciência e arte, porque a tomada de decisão racional e baseada em evidências geralmente envolve a escolha de uma alternativa entre um conjunto de escolhas racionais”. Cabe reconhecer, a propósito, que escolhas racionais não são política e ideologicamente neutras, mas informadas por valores e crenças que lhes conferem diferentes racionalidades, todas legítimas.

As potencialidades e limitações deste artigo são as inerentes à abordagem da SPBE. Apresenta-se, tacitamente, potencialidades para a compreensão do papel que a fluoretação vem exercendo nos 40 anos de sua implementação na cidade. Suas limitações derivam do fato de se ocuparem de uma intervenção de saúde pública que vem se desenvolvendo há quatro décadas. O artigo explora o período da primeira dessas quatro décadas, discutindo os achados com a literatura pertinente, e apresenta possibilidades para a compreensão das três últimas décadas, situando a referida intervenção no contexto histórico em que se desenvolveu e apresenta o problema que sua eventual interrupção pode acarretar.

## CONCLUSÃO

Considerando as dificuldades persistentes de acesso a cuidados odontológicos assistenciais na cidade de São Paulo e, diante do risco de interrupção da implementação da fluoretação em São Paulo, trazido pelo debate contemporâneo sobre o tema, no Brasil e no mundo, é cabível indagar, como expusemos neste artigo, sobre o impacto dessa eventual interrupção sobre os níveis populacionais de cárie dentária.

As pressões pela descontinuidade da fluoretação da água na cidade de São Paulo e em outras localidades brasileiras^37^, com base em opiniões desprovidas de evidência científica, devem ser rejeitadas por tomadores de decisão no âmbito das políticas públicas. Com essa finalidade, a perspectiva da SPBE, apresentada neste artigo, pode contribuir para fundamentar a continuidade dessa medida preventiva na cidade.

Como tecnologia de saúde pública, a fluoretação da água não é um dogma^38^, nem está fechada à pesquisa científica. Deve ser avaliada, com o devido rigor científico e, se necessário, pode-se não a utilizar – como fazem alguns países. Mas uma decisão dessa natureza deve ser tomada levando-se em conta a sua complexidade. Motivações religiosas, econômicas, epidemiológicas e sanitárias precisam ser bem analisadas como fundamento da decisão.

## Data Availability

O estudo foi conduzido a partir de dados apurados de bancos de dados disponibilizados publicamente pelo Ministério da Saúde. Qualquer interessado pode ter acesso às referidas bases de dados, mediante solicitação à Coordenação-Geral de Saúde Bucal do Ministério da Saúde, por meio do endereço eletrônico: cosab@saude.gov.br.
